# Editorial: Human-centered design for HRI in manufacturing

**DOI:** 10.3389/frobt.2026.1773276

**Published:** 2026-02-20

**Authors:** Jose Antonio Mulet Alberola, Ganix Lasa Erle

**Affiliations:** 1 Universita degli Studi di Brescia, Brescia, Italy; 2 Istituto di Sistemi e Tecnologie Industriali Intelligenti per il Manifatturiero Avanzato Consiglio Nazionale delle Ricerche, Milan, Italy; 3 Design Innovation Center (DBZ), Mondragon Unibertsitatea, Arrasate, Spain

**Keywords:** hcd, HRI, HRI in manufacturing, human-centered design, human-robot interactions

New manufacturing paradigms are supported by collaborative systems that emphasize the value created through individual interactions and tasks rather than traditional job roles. As part of this development and new research lines, this Research Topic aims to capture the importance of the human factor in designing and developing new technology that interacts with humans and whose functionality leverages the adoption of the human-centered design concept.

In terms of physical safety, (Han et al.) studies the power and force limiting (PFL) mode according to ISO/TS 15066. Measurements are taken on different trials to identify a biomechanical limit that ensures collision safety in Human-Robot Interactions (HRI). The analysis is limited by impact areas and subjects but shows promising evaluation criteria for the evaluation of threshold forces.

The concept of flow is introduced in (Prajod et al.) as the optimal state in human-robot collaboration that mainly improves both task performance and user experience. It is characterized by various factors such as intense focus, immersion, and a significant sense of control. This three-channel flow model correlates flow states, boredom, and anxiety. The study shows that task complexity and robot behaviors in collaborative tasks affects flow status and influences the level of challenge experienced by users. Both factors are suggested to be adjusted dynamically for an improved experience.

To adopt a more natural behavior in HRI (Lavit Nicora et al.), explores users’ direct eye-gaze as a cognitive action that shows a willingness to interact. Most participants tended to look directly at the collaborative robot when triggering the joint activity but kept looking at the robot throughout the entire collaborative action or assembly cycle. Results demonstrate that natural social habits exhibited in human-human interactions may also be used when interacting with robots in a collaborative manner. However, further technical improvements need to be made when deploying such a system to improve performance while ensuring natural user feelings throughout the interaction.

As these interactions evolve and expand into more industrial scenarios, granular patterns emerge, revealing subtle differences between interaction types. This emphasizes the variety and variability of interactions, providing a valuable foundation for the understanding of subtle differences during interactions. Although not completely characterized (Heuermann et al.), reveals that automatic clustering can reliably and reproducibly find meaningful, distinct interaction forms in different scenarios based on spatial and temporal behaviors and outperform more classical clustering approaches.

These promising studies suggest further work needs to be done not only in terms of physical interactions but also in social ones. The editors and coordinators of this Research Topic highly encourage the further study of HRI in real industrial scenarios by the evaluation and analysis of all conditions, with the aim to create more dynamic interaction‐based relationships that leads to enhanced performance and wellbeing. The presented works support the importance of human factors to design and develop new technology that closely interacts with humans and whose functionality leads to an increase in performance, safety, and efficiency while avoiding misuse and/or disuse as manufacturing systems evolve.

